# Comparison of p27 Gene Expression of Promastigote and Amastigote Forms of *Leishmania major* (MRHO/IR/75/ER) by Real-time RT-PCR

**Published:** 2018

**Authors:** Samira ELIKAEE, Mehdi MOHEBALI, Hamid ESLAMI, Sassan REZAEI, Hamid Reza NAJAFIAN, Elham KAZEMI-RAD, Hossein KESHAVARZ, Mohammad Reza ESHRAGHIAN, Homa HAJJARAN, Mohammad Ali OSHAGHI, Sara AYAZIAN MAVI

**Affiliations:** 1. Dept. of Medical Parasitology and Mycology, School of Public Health, Tehran University of Medical Sciences, Tehran, Iran; 2. Center for Endemic Parasites of Iran, Tehran University of Medical Sciences, Tehran, Iran; 3. Dept. of Medical Biotechnology, School of Advanced Technologies in Medicine, Tehran University of Medical Sciences, Tehran, Iran; 4. Dept. of Parasitology, Pasteur Institute of Iran, Tehran, Iran; 5. Dept. of Biostatistics and Epidemiology, School of Public Health, Tehran University of Medical Sciences, Tehran, Iran; 6. Dept. of Medical Entomology and Vector Control, School of Public Health, Tehran University of Medical Sciences, Tehran, Iran

**Keywords:** p27 gene, Real-time RT-PCR, *Leishmania major*, Amastigote, Promastigote

## Abstract

**Background:**

Cutaneous leishmaniasis (CL) is one of the world health problems. *Leishmania major* is the etiological agent of zoonotic cutaneous leishmaniasis (ZCL). Promastigote and amastigote are two morphological forms of *Leishmania* parasites that express different proteins and p27 is an important gene encoding cytochrome c oxidase (COX) component. P27 gene expresses a 27 kDa protein that essential in ATP synthesis. This study aimed to compare p27 gene expression in promastigote and amastigote forms in Iranian strain of *L. major* (MRHO/IR/75/ER).

**Methods:**

This study was conducted in 2015. Clinical isolates of CL patients from north, center, west and south parts of Iran were collected and identified by PCRRFLP. After RNA extraction of promastigotes and amastigotes and cDNA synthesis, the expression level of p27 gene was compared by real-time RT-PCR.

**Results:**

By comparison of expression level between amastigote and promastigote forms of Iranian strain of *L. major*, up-regulation of p27 gene (2.73 fold) was observed in amastigotes. Moreover, there was no significant difference in p27 gene expression between *L. major* isolates.

**Conclusion:**

p27 gene and protein can be considered as a target in recombinant vaccine production and treatment process.

## Introduction

Intracellular protozoan parasites of the genus *Leishmania* are the cause of wide spectrum of diseases that called leishmaniasis (1). Overall, 350 million people in 98 countries are at risk. 1300000 new cases, 30-40000 death per year and 1974000 DALYs (Disability-Adjusted Life Years) were estimated (2).

Zoonotic cutaneous leishmaniasis (ZCL), caused by *L. major*, is endemic in 17 out of 31 provinces of Iran (3). *Leishmania* is a dimorphic parasite, promastigote form which exists in the sand fly vector and amastigote form that lives inside infected mammalian macrophages (4). Amastigotes change their gene expression levels to adapt themselves with environment inside the macrophages (5, 6). Some of these changes could be targeted to prevent subsequent infections (4).

Increased mitochondrial activity may involve in maintaining the parasite inside macrophages (7, 8). The inner mitochondrial membrane protein complexes have been implicated in electron transfer to oxygen (9, 10). Cytochrome c oxidase (complex IV) is recognized in both *Trypanosoma* and *Leishmania* (11, 12). This complex is consisting of more than 14 subunits that its function is still not well known (13).

This is the first study that describes the expression level of a gene encoding a 27 kDa mitochondrial membrane protein (p27), a subunit of the active COX in amastigote of *L. major* compared to promastigote by quantitative molecular method. The results of this study will inform about a target for drug discovery and vaccine development effort.

### Materials and Methods

#### Clinical samples and culture

This study was conducted in 2015. Four *Leishmania* sp. were isolated from CL cases from north (Golestan Province), center (Tehran Province), west (Ilam Province) and south (Fars Province) of Iran. All isolates were cultured in NNN medium. For mass production, promastigotes were passaged in RPMI 1640 of Biosera, U.K company (Cat No. LMR16381500, Lot No. 014BS371) supplemented with 10% fetal calf serum (FCS) (Gibco, Germany #10270) and 100U/ml penicillin and 100μg/ml streptomycin (Gibco, Germany#1078514) at 23–25 °C (14).

### DNA extraction and characterization with PCR-RFLP

Total DNA from promastigote forms were extracted with the High Pure PCR Template Preparation Kit (Roche-Germany). Then with using of PCR-RFLP method and N-acetylglucosamine-1-phosphate transferase (NAGT) gene that described previously, *L. major* was confirmed in all isolates (14).

### Cell culture and amastigote isolation

**Fig. 1:**
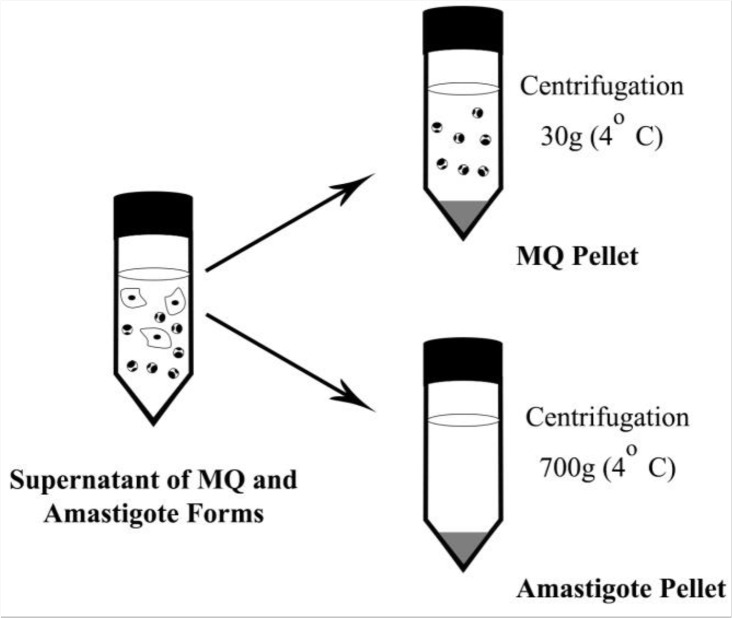
Schematic graphic of collection of the liberated amastigotes from peritoneal macrophages (MQs) of BALB/c mice by differential centrifugation

Peritoneal macrophages were obtained 48 h after intraperitoneal injection of 1 ml of 3% thioglycollate broth (Sigma-Aldrich) by washing the peritoneal cavity of BALB/c mice. Macrophage viability was determined by Trypan Blue stain and 2×10^5^ cells were cultured on 48-well plates (Nunc, Denmark) (15, 16). Cultures were done in 500 μl supplemented RPMI 1640 medium containing 10% FCS and 100U/ml penicillin and 100μg/ml streptomycin. After 20 h culture, nonadherent cells were discarded. Adherent cells were infected with stationary-phase promastigotes of *L. major* at 10:1 parasite/macrophage ratio for 20 h in condition of 37 °C and 5% CO_2_. Then extracellular parasites were washed and cells treated with fresh medium for 96 h (15, 17, 18). Amastigotes were exited from macrophages by using aspiration with 22 gauge needle and collected with differential centrifugation; a 30 gr centrifugation to separate the macrophages and a 700 gr centrifugation to obtain the amastigote pellet (Fig. 1) (19).

### RNA isolation and cDNA synthesis

By using of Tripure reagent accordance with the manufacturer’s method (Roch, Mannheim, Germany), total RNA was isolated from 10^8^ amastigotes and promastigotes of 4 *L. major* isolates, separately, when they were in early stationary phase. The concentration of RNA was determined by spectrophotometer with absorbance at 260 nm (NanoDrop, USA). DNA genomic contamination was eliminated with DNase1 (Qiagen, Hilden, Germany) and 45 min incubation at 37 °C. Total RNA was reverse transcribed using the cDNA Synthesis Kit (Roche, Germany#13030821)) according to its instructions (20).

### Real-Time RT-PCR

Real-Time PCR assay was done on the p27 gene as a target and Alpha-tubulin as a housekeeping gene. The primers were designed by Primer 3 software ver. 0.4.0 (http://frodo.wi.mit.edu/). p27 primers including forwarding (5- GGACCCGATCCGTGAGATATAC) and reverse (5-GTTGAGAGGACGGATGTTGC) were used for amplification of a 145bp fragment in the experiments. For this purpose Applied Biosystem step one (ABI company) with 12 μl of 2 x Master Mix Green High ROX^TM^ (AMPIQON, Denmark # 15H801), 1 μl of cDNA, and 100 nM primers in a final volume of 20 μl were used.

The PCR condition was as follows: an initial denaturation at 95 °C for 3 min, 40 cycles of 10 sec at 95 °C and 32 sec at 62 °C followed by a melt curve analysis using temperature increments of 0.3 °C every 30 sec to ascertain amplification of the expected product.

The relative p27 gene expression obtained by comparing the cycle thresholds (CTs) of the target gene with Alpha-tubulin as a housekeeping gene using the relative expression software tool (REST, https://www.gene-quantification.de/rest.html) (21).

### Data Analysis

The relative gene expression value of p27 gene in the *L. major* isolates was normalized to the internal control gene (Alpha-tubulin) using the 2^−ΔΔct^ method (22, 23). The significance of differences was determined by the relative expression software tool (REST, https://www.gene-quantification.de/rest.html). All experiments were done at least three times and the results are reported as the mean ± standard deviations (SDs). The expression ratio results of the target gene were tested for significance by a Pair Wise Fixed Reallocation Randomization Test and plotted using standard error (SE) estimation via a complex Taylor algorithm, calculated by REST. Samples with a *P*-value of <0.001 were considered significantly different among the groups (21).

## Results

### NAGT PCR-RFLP

**Fig. 2:**
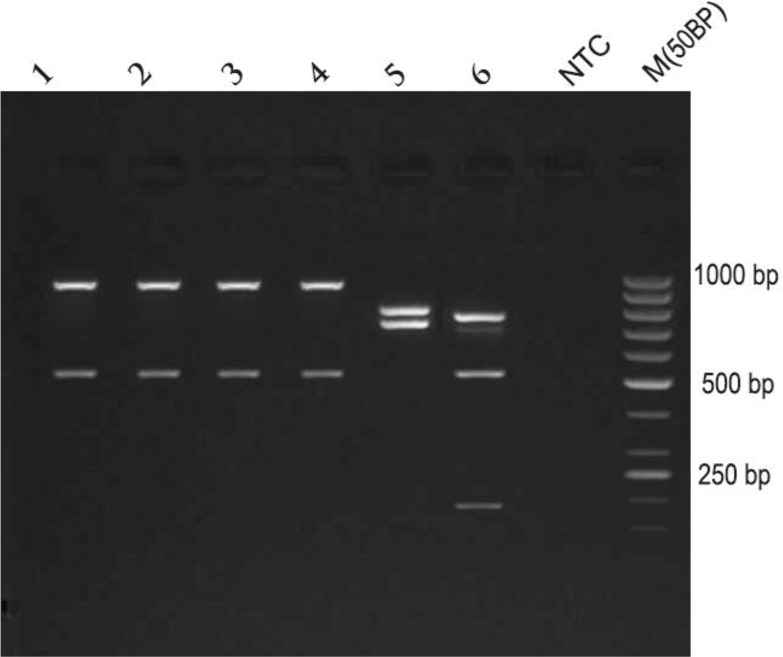
Patterns of NAGT amplicons digestion with AccI enzyme in test isolates and standard *Leishmania* strains. Lane 1–4: *L. major* (lane 4 also as a standard strain of *L. major*). Lane 5: *Leishmania tropica* (MHOM/IR/01/yaza). Lane 6: *Leishmania infantum* (MCAN/IR/07/Moheb-gh). Lane 7: None template control (NTC). Lane 8: Marker (50 bp)

After DNA extraction, all samples were characterized by PCR-RFLP and NAGT gene. After digestion with AccI enzyme, all isolates and the reference strain exhibited two bands (950 bp, 500 bp) on agarose gel which correspond to *L. major* (Fig. 2).

### cDNA synthesis

In order to evaluate the integrity of synthesized cDNA, PCR was conducted using Alpha-tubulin primers as housekeeping gene. When clinical samples gave single band of 119 bp, PCR result was considered positive.

### Real-Time RT-PCR analysis

**Fig. 3:**
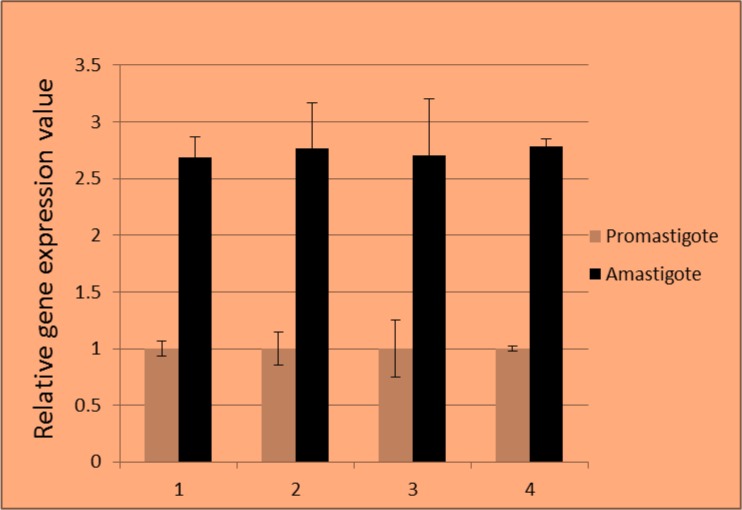
Comparison of p27 gene expression level in promastigote and amastigote forms of *L. major* isolates

Real-time RT-PCR was used to determine and compare the relative expression of p27 gene in amastigotes and promastigotes of *L. major* isolates from different part of Iran. Fig. 3 shows a significant up-regulation of p27 gene in amastigotes of each *L. major* isolates (*P*<0.001). The average mRNA expression of p27 gene in amastigotes was 2.73 fold of its expression in promastigotes. Moreover, there was no significant difference in p27 gene expression between *L. major* isolates.

## Discussion

p27 gene expresses a 27 kDa-protein. This protein as a part of the active COX complex involved in oxidative phosphorylation that intracellular parasites need it to survive. When amastigotes, reside in acidic phagolysosomes, experience near-constant temperatures, and encounter host factors that subject them to oxidative, proteolytic, and metabolic stresses (24–27). mRNA of *L. major* p27 gene is more expressed in amastigote than promastigote forms. Overexpression of this protein as a part of mitochondrial structure confirms parasite answer to oxidative stress. Similarly, a previous study on transcriptome analysis of *Leishmania donovani* (*L. donovani*) using genomic microarrays showed more abundant expression of p27 gene in *L. donovani* amastigotes (27). In addition, the results of another study on *L. donovani* stage-specific mitochondrial membrane protein, p27gene also revealed more expression in amastigotes and metacyclic promastigotes than in procyclic promastigotes (4). Other documents reported amastins (28), HASPA1,2 (29), SCG5 and SCG7 which are members of the phosphoglycan beta 1,3 galactosyltransferase gene family (30) as *L. major* amastigote-upregulated genes and also his-tones (31, 32), the glucose transporter GT2 (33) and the leishmanolysin GP63 (also called MSP) (34, 35) as differentially expressed genes in promastigotes.

Gene expression had not significant difference in *L. major* isolates of different geographical areas, although in promastigotes significantly less than amastigotes. COX complex had fundamental function in *L. major* promastigote and amastigote forms. Requirement of active COX complex was confirmed in proliferation of *Leishmania* promastigotes and amastigotes (4, 10, 11). p27 as a part of COX complex may be an appropriate target for control and prevention of *L. major* amastigotes proliferation in host cells. However, further studies are needed to define precise biological function for the mentioned protein in the process of vaccination and treatment.

## Conclusion

Analysis of p27 gene expression in *L. major* promastigote and amastigote forms by real-time RT-PCR demonstrated up-regulation of p27 gene expression in amastigotes of *L. major*. Therefore, it may have an important role in proliferation, virulence, pathogenesis, and survival of amastigotes in host.
